# Time to be brave: is educating surgeons the key to unlocking the potential of randomized clinical trials in surgery? A qualitative study

**DOI:** 10.1186/1745-6215-14-S1-P2

**Published:** 2013-11-29

**Authors:** Shelley Potter, Nicola Mills, Simon Cawthorn, Jane Blazeby

**Affiliations:** 1 Centre for Surgical Research, School of Social and Community Medicine, University of Bristol, Bristol, UK; 2 Breast Care Centre, North Bristol NHS Trust, Bristol, UK; 3 Division of Surgery, Head and Neck, University Hospitals Bristol NHS Foundation Trust, Bristol, UK

## Background

Well-designed randomised clinical trials (RCTs) provide the optimal evidence to inform decision-making and should be the default for evaluating surgical procedures. Such trials can be challenging and surgeons' preferences may influence whether trials are initiated, successfully conducted and the results accepted. Preferences are particularly problematic when there are several different surgical options and surgeons' views play a key role in procedure selection. The basis of such preferences, however, have never been formally explored. The aim of this study was therefore to use qualitative methods to explore surgeons' preferences and how they may influence the feasibility of surgical randomised clinical trials (RCTs).

## Methods

Semi-structured qualitative interviews were undertaken with a purposive sample of 35 professionals practicing at 15 centres across the UK. Interviews were transcribed verbatim and analysed using the constant comparative technique of grounded theory. Sampling, data collection and analysis were conducted concurrently and iteratively until data saturation was achieved.

## Results

Professionals did not accept randomization as a method of treatment allocation when they lacked equipoise for treatment options. The underlying reasons for limited equipoise were limited appreciation of the methodological weaknesses of data from non-randomised studies and little understanding of pragmatic trial design. Beliefs regarding the value of RCTs for generating high-quality data to change or inform practice were therefore not widely held.

## Conclusion

There is a need to help surgeons understand evidence and bias. Current NIHR/MRC investment into education and infrastructure for RCTs combined with strong leadership may address these issues.

